# Health Insurance Is Associated with Decreased Odds for Undiagnosed Prediabetes and Type 2 Diabetes in American Adults

**DOI:** 10.3390/ijerph17134706

**Published:** 2020-06-30

**Authors:** Sean Mahoney, Adam Bradley, Logan Pitts, Stephanie Waletzko, Sheria G. Robinson-Lane, Timothy Fairchild, Donna J. Terbizan, Ryan McGrath

**Affiliations:** 1 Department of Health, Nutrition, and Exercise Sciences, North Dakota State University, Fargo, ND 58108, USA; sean.mahoney@ndsu.edu (S.M.); adam.bradley@ndsu.edu (A.B.); logan.pitts@ndsu.edu (L.P.); stephanie.waletzko@ndsu.edu (S.W.); d.terbizan@ndsu.edu (D.J.T.); 2 School of Nursing, University of Michigan, Ann Arbor, MI 48109, USA; grices@med.umich.edu; 3 Centre for Molecular Medicine & Innovative Therapeutics, Murdoch University, 6150 Perth, Australia; T.Fairchild@murdoch.edu.au

**Keywords:** delivery of healthcare, healthcare disparities, mandatory testing, socioeconomic factors

## Abstract

Over a third of adults in the United States have prediabetes, and many of those with prediabetes will progress to type 2 diabetes within 3–5 years. Health insurance status may factor into a proper diagnosis of prediabetes and diabetes. This study sought to determine the associations between health insurance and undiagnosed prediabetes and diabetes in a national sample of American adults. Publicly available data from 13,029 adults aged 18–64 years from the 2005–2016 waves of the National Health and Nutrition Examination Survey were analyzed. Health insurance type (Medicaid, Private, Other, None) was self-reported. Prediabetes and diabetes status were assessed with measures of self-report, glycohemoglobin, fasting plasma glucose, and two-hour glucose. Covariate-adjusted logistic models were used for the analyses. Overall, 5976 (45.8%) participants had undiagnosed prediabetes, while 897 (6.8%) had undiagnosed diabetes. Having health insurance was associated with decreased odds ratios for undiagnosed prediabetes: 0.87 (95% confidence interval (CI: 0.79, 0.95)) for private insurance, 0.84 (CI: 0.73, 0.95) for other insurance, and 0.78 (CI: 0.67, 0.90) for Medicaid. Moreover, having private health insurance was associated with 0.82 (CI: 0.67, 0.99) decreased odds for undiagnosed diabetes. Health insurance coverage and screening opportunities for uninsured individuals may reduce prediabetes and diabetes misclassifications.

## 1. Introduction

The health consequences and increasing prevalence of type 2 diabetes are substantial public health burdens at both individual and population levels. For example, type 2 diabetes reduces quality of life, increases multimorbidity risk, and accelerates time to mortality [1,2]. These complications also have pronounced economic implications on healthcare systems, especially as the prevalence of those with type 2 diabetes continues to grow [1,3,4,5]. As the American population ages and the prevalence of individuals with type 2 diabetes continues to rise, projections place the number of diagnosed type 2 diabetes cases in the United States around 39 million by the year 2050 [1].

Type 2 diabetes is preceded by prediabetes, a condition that is characterized by either impaired fasting glucose or glucose tolerance [6]. In the United States, over a third of adults have prediabetes, with 25% developing type 2 diabetes within 3–5 years [7,8] and 70% developing type 2 diabetes within their lifetime [9]. Early lifestyle alterations and pharmacologic interventions in those with prediabetes can delay or even prevent the onset of type 2 diabetes [6,7,10]. For example, early lifestyle interventions may reduce the incidence of type 2 diabetes in high-risk populations [7]. Unfortunately, the vast majority of Americans with prediabetes are unaware of their condition and interventions are therefore not implemented [11]. Further, there is a low level of awareness regarding prediabetes across all demographic groups [11].

Early screening for prediabetes provides an opportunity for the restoration of euglycemia and prevention of type 2 diabetes [12]. However, few Americans with prediabetes are aware of having the condition, and the awareness of prediabetes remains low in the general population [11]. Disparities in screening opportunities associated with health insurance coverage may limit opportunities for awareness of prediabetes status and ultimately proactive intervention [13,14,15]. Therefore, it is possible health insurance status plays an important role in both the awareness and diagnosis of prediabetes and type 2 diabetes. This study sought to determine the associations between health insurance status and undiagnosed prediabetes and type 2 diabetes in American adults.

## 2. Materials and Methods

### 2.1. Participants

A secondary analysis of publicly available data from 15,036 Americans aged 18–64 years with measures of diabetes (objective and self-reported) and health insurance type from the 2005–2006, 2007–2008, 2009–2010, 2011–2012, 2013–2014, and 2015–2016 cross-sectional waves of the National Health and Nutrition Examination Survey (NHANES) was conducted. The NHANES is a program of studies designed to assess the health and nutrition status of American children, adolescents, adults, and older adults [16]. Mobile examination centers traveled to various locations throughout the United States. Trained personnel completed health interviews in participant residences using computer-aided interview systems, and participants also visited mobile examination centers for enhanced examinations [17]. 

Oversampling occurred for those aged at least 60 years, non-Hispanic Asians, non-Hispanic Blacks, and Hispanics to produce reliable data that were representative of all ages and ethnicities in the United States [16]. Overall interview response rates were ≥58.7% for each wave of the NHANES included in our analyses [18]. The NHANES utilizes a complex, four-stage probability sampling design to generate a representative sample of non-institutionalized Americans. Written informed consent was provided by participants, and NHANES protocols (Protocol #2005-06; Protocol #2001-17) were approved by the National Center for Health Statistics Research Ethics Review Board.

### 2.2. Measures

Prediabetes and type 2 diabetes were assessed with measures of glycohemoglobin (HbA1c), fasting plasma glucose, and two-hour glucose (oral glucose tolerance test). A phlebotomist collected blood samples from participants according to standardized protocols at mobile examination centers. Changes to the laboratory methods such as instrumentation and calibration procedures occurred during the waves of the NHANES that we evaluated; however, these methodological changes had negligible effects on the measures, and adjustments in calibrations were not advised across these NHANES waves [19]. Recommendations from the American Diabetes Association were used for determining prediabetes and type 2 diabetes [20]. Those with prediabetes had either glycohemoglobin between 5.7% and 6.4%, fasting plasma glucose between 100 mg/dL and 125 mg/dL, or two-hour glucose between 140 mg/dL and 199 mg/dL. Similarly, those with type 2 diabetes had either glycohemoglobin ≥6.5%, fasting plasma glucose ≥126 mg/dL, or two-hour glucose ≥200 mg/dL.

Respondents told interviewers if a healthcare provider had ever diagnosed them with prediabetes or type 2 diabetes. Those who responded “borderline” for the self-report type 2 diabetes diagnosis inquiry were classified as having prediabetes. Participants were considered as having undiagnosed prediabetes if they responded as not having prediabetes or diabetes, but either their glycohemoglobin, fasting plasma glucose, or two-hour glucose measures indicated they had prediabetes. Likewise, participants were considered as having undiagnosed type 2 diabetes if they responded as not having type 2 diabetes but either their glycohemoglobin, fasting plasma glucose, or two-hour glucose measures indicated they had type 2 diabetes.

Interviewers asked respondents if they were covered by health insurance or some other type of healthcare plan. Those suggesting they had health insurance or another similar type of healthcare plan revealed if they were insured privately, with Medicaid, or had another type of healthcare plan (i.e., other). Respondents covered by Medicare were not considered for our investigation because older adults were excluded a priori.

Age, sex, and race/ethnicity were self-reported. Standing height was measured with a fixed stadiometer, and body mass was collected with a digital scale (Mettler-Toledo International Inc.; Columbus, OH). Body mass index (BMI) was calculated as body mass in kilograms divided by height in meters squared (kg/m^2^). Those with a BMI of ≥30 kg/m^2^ were considered obese [21]. Self-rated health was measured with a single-item, wherein respondents perceived their health as either “excellent”, “very good”, “good”, “fair”, or “poor”. Highest level of educational achievement was self-reported and categorized as not completing high school or completing high school/equivalent exam. Participants were considered below the poverty threshold if their ratio of family income to poverty was less than 1.0 [22]. Respondents told interviewers where they most often go for healthcare. Places of healthcare were categorized as either “emergency room, outpatient department, other”, “clinic or health center”, “doctor’s office or health maintenance organization”, or “none”. Protocols for each variable at specific waves of the NHANES are available on their website (https://www.cdc.gov/nchs/nhanes/index.htm). 

### 2.3. Statistical Analyses

All analyses were executed with SAS 9.4 software (SAS Institute; Cary, NC, USA). A logistic regression model analyzed the associations of health insurance type and undiagnosed prediabetes. Another logit model determined the associations of healthcare insurance type and undiagnosed type 2 diabetes. Cases of undiagnosed prediabetes and type 2 diabetes were then collapsed, and a third logistic model examined the associations of health insurance type and any undiagnosed prediabetes or type 2 diabetes. 

Separate logit models were again performed to determine the associations between health insurance type and undiagnosed prediabetes, undiagnosed type 2 diabetes, and any undiagnosed prediabetes or type 2 diabetes by sex and age group (aged 18–44 years and 45–64 years). All of the logistic models were adjusted for age, sex (when applicable), race/ethnicity, obesity, self-rated health, poverty, educational achievement, and place of healthcare. Those with no health insurance were also the reference group for each logit model. The covariates were pre-specified by the investigators because they were thought of as being influential for our findings. 

Secondary analyses were conducted to determine the associations between health insurance type and undiagnosed prediabetes, undiagnosed type 2 diabetes, and any undiagnosed prediabetes or type 2 diabetes by educational achievement (not completing high school, completing high school/equivalent exam) and race/ethnicity (white, not white). Individual logistic models analyzed these associations and the same covariates used in our principal analyses were also used for these secondary analyses. The findings from our secondary analyses were presented as an appendix because the secondary analyses were not part of our a priori study purpose. An alpha level of 0.05 was used for all analyses.

## 3. Results

After exclusions for missing covariates (*n* = 2007), the analytic sample included 13,029 participants and their descriptive characteristics are presented in Table 1. Overall, participants were 44.6 ± 12.8 years of age, and 5976 (45.8%) had undiagnosed prediabetes, while 897 (6.8%) had undiagnosed type 2 diabetes. To make comparisons between the health insurance groups for the descriptive characteristics, means and 95% confidence intervals (CIs) are provided in Table A1. Those with no health insurance had significantly higher proportions of undiagnosed prediabetes or diabetes (57.6%; CI: 56.0%, 59.2%) compared to adults with private health insurance (51.2%; CI: 50.0%, 52.4%), Medicaid (47.2%; CI: 44.3%, 50.2%), or other health insurance (51.7%; CI: 49.1%, 54.3%).

Table 2 presents the results for the associations of health insurance type and undiagnosed prediabetes and type 2 diabetes. Having health insurance was associated with decreased odds ratios (OR) for undiagnosed prediabetes: 0.87 (CI: 0.79, 0.95) for private insurance, 0.84 (CI: 0.73, 0.95) for other insurance, and 0.78 (CI: 0.67, 0.90) for Medicaid. Private insurance was also associated with decreased odds for undiagnosed diabetes (OR: 0.82; CI: 0.67, 0.99), but not Medicaid (OR: 0.86; CI: 0.65, 1.13) nor other health insurance (OR: 0.83; CI: 0.65, 1.07). Moreover, private health insurance (OR: 0.82; CI: 0.74, 0.90), other health insurance (OR: 0.80; CI: 0.70, 0.91), and Medicaid (OR: 0.75; CI: 0.65, 0.86) decreased the odds ratios for undiagnosed prediabetes and diabetes.

In males, private insurance (OR: 0.85; CI: 0.75, 0.97), other insurance (OR: 0.82; CI: 0.68, 0.98), and Medicaid (OR: 0.66; CI: 0.53, 0.83) decreased the odds ratios for undiagnosed prediabetes (Table 3). Although there were non-significant results for the associations of health insurance and undiagnosed type 2 diabetes in males, private health insurance (OR: 0.81; CI: 0.70, 0.92), other health insurance (OR: 0.76; CI: 0.63, 0.91), and Medicaid (OR: 0.64; CI: 0.51, 0.80) all were associated with decreased odds for undiagnosed prediabetes or type 2 diabetes. In females, private health insurance (OR: 0.85; CI: 0.74, 0.99) decreased the odds ratios for having undiagnosed prediabetes or diabetes, but not Medicaid or other health insurance. For those aged 18–44 years, only private insurance decreased the odds ratios for undiagnosed type 2 diabetes (OR: 0.67; CI: 0.48, 0.93) and any undiagnosed prediabetes or type 2 diabetes (OR: 0.83; CI: 0.72, 0.96). However, private health insurance was not associated with decreased odds for having undiagnosed prediabetes. In those aged 45–64 years, Medicaid and other health insurance decreased the odds ratios for undiagnosed prediabetes (Medicaid OR: 0.70; CI: 0.57, 0.87; other health insurance OR: 0.83; CI: 0.70, 0.99) and any health insurance was associated with decreased odds for any undiagnosed prediabetes or type 2 diabetes (private health insurance OR: 0.85; CI: 0.74, 0.98; other health insurance OR: 0.80; CI: 0.67, 0.95; Medicaid OR: 0.69; CI: 0.56, 0.85). However, health insurance was not associated with undiagnosed type 2 diabetes in adults 45–64 years of age.

Table A2 reveals the results for the associations of health insurance type and undiagnosed prediabetes and type 2 diabetes by educational achievement and race/ethnicity. Differential associations existed when stratifying the analyses by these factors. 

## 4. Discussion

The principal results of this investigation revealed that health insurance was associated with decreased odds for undiagnosed prediabetes or diabetes in American adults. Specifically, those with private insurance, other insurance, and Medicaid had 13%, 16%, and 22% decreased odds for undiagnosed prediabetes, respectively. Private health insurance was associated with 18% decreased odds for type 2 diabetes. Moreover, those with private insurance, other insurance, and Medicaid had 18%, 20%, and 25% decreased odds of having either undiagnosed prediabetes or diabetes, respectively. Sex and age also factored into the associations of health insurance type on undiagnosed prediabetes and type 2 diabetes. Our results show that those with no access to healthcare may not be aware of their prediabetes or type 2 diabetes condition. These findings underscore the critical role of health insurance for the diagnosis of prediabetes and diabetes in American adults.

Early detection and identification of prediabetes and type 2 diabetes are crucial for slowing the progression of the disease and management of comorbidities [13,14,23,24]. In a 10-year longitudinal follow-up of newly diagnosed individuals with type 2 diabetes, those identified with unstable HbA1c trajectories had increased risk of microvascular events compared to those that had stable HbA1c [24]. In our study, large proportions of participants were classified as either having undiagnosed prediabetes or type 2 diabetes despite health insurance type. The large proportions of those who are unaware of their prediabetes or type 2 diabetes are concerning because these individuals are not actively managing their glucose levels and living with elevated HbA1c. Individuals who are uninsured are particularly susceptible, as they lack access to screening opportunities and pharmaceutical treatment options (i.e., metformin). Considering that in 2018, about 27.5 million (~8.5%) Americans did not have health insurance, approximately 13 million Americans could be living with prediabetes or type 2 diabetes and have not been diagnosed with the condition nor is the condition being managed [25]. With the growing prevalence of obesity and the ageing population [1], the number of people living with undiagnosed type 2 diabetes and prediabetes is likely to grow. 

Risk factors for prediabetes and type 2 diabetes include several modifiable and non-modifiable characteristics [26,27,28]. Age and BMI are simple and easy metrics to dictate screening opportunities and are included in nearly all screening guidelines. For example, the United States Preventative Services Task Force (USPSTF) recommends screening for abnormal blood glucose levels and type 2 diabetes in adults aged 40–70 years who are overweight or obese. The American Diabetes Association recommends screening for prediabetes beginning at age 45 for all adults and screening for type 2 diabetes in all asymptomatic adults who are overweight or obese. Only the American Academy of Clinical Endocrinologists calls for screening of all adults aged at least 45 and younger adults with any risk factor, regardless of body weight [27]. Incidentally, a recent investigation that utilized 1988–2012 NHANES data reported an 8.3% increase in prevalence rates for prediabetes among individuals aged at least 20 years within a healthy BMI range [8,23]. Further, in a similar investigation, 66% of respondents met the American Diabetes Association’s diabetes screening criteria; however, only 46.2% reported receiving any form of screening such that 3.7% were classified as having undiagnosed type 2 diabetes and 36.3% were classified as having undiagnosed prediabetes [29]. O’Brien et al. [30] revealed only half of American adults with dysglycemia would be identified following USPSTF screening criteria and that more rigorous diabetes risk factors should be included in screening efforts to promote early detection and intervention in high-risk groups.

The findings of this investigation showed that men with any health insurance type had decreased odds of undiagnosed prediabetes. This could be a result of current screening criteria and differences in risk factors for prediabetes among men and women. In a previous population-based cohort study, it was reported that among men and traditional anthropometric measurements (i.e., BMI) were stronger risk markers for predicting type 2 diabetes compared to combined metabolic indices (e.g., visceral adiposity index, lipid accumulation product, and product of triacylglycerol and glucose) [31]. In contrast, the opposite was found to be true for women [31]. Additionally, in a similar investigation, predicted fat mass was a consistently stronger risk marker for the development of type 2 diabetes compared to BMI for both men and women [32]. Prevailing female risk factors may require more invasive screening procedures which are not in routine clinical use, and therefore, females with these risk factors may not be referred to screening; whereas the risk factors for males, such as BMI, are part of routine clinical screening and are easily identified, resulting in correct referral of males for the screening of type 2 diabetes or prediabetes [33]. This is also supported in our investigation with the Medicaid health insurance group having the highest proportion of obese respondents yet the lowest odds for undiagnosed prediabetes among men. 

In addition, older individuals with any form of health insurance had decreased odds for undiagnosed prediabetes or type 2 diabetes, but in those aged 18–44, only private health insurance showed decreased odds. Interestingly, there appears to be an increasing “gap” between uninsured and insured individuals as age increases, likely due to diabetes guidelines recommending screening start at around age 45. However, due to age-related changes in skeletal muscle mass and height, these measures may not adequately quantify body fat, the stronger predictor for type 2 diabetes risk. Additionally, overall adiposity, central adiposity, and weight gain during middle age are associated with a type 2 diabetes risk among the elderly [34]. Current screening criteria using traditional anthropometric measurements may benefit male individuals at risk for diabetes but are not robust enough to identify all individuals at risk. This is especially true for women and older adults, where the association between BMI and diabetes remains robust.

Similarly, diabetes awareness is low among at-risk individuals, compounding the previously highlighted issue of many adults living with prediabetes not being identified through existing screening efforts [5]. In 2010, a large proportion of adults in the United States had undiagnosed prediabetes [35,36], and only 46% of adults with risk factors for prediabetes and type 2 diabetes received screening [27]. Prevention of type 2 diabetes progression and improved glycemic control require regular interaction between healthcare providers and patients. Communication of risk information is a fundamental step in health care interventions [23,37,38]; however, patients do not often receive sufficient information from healthcare providers on disease awareness and the steps they can take to prevent further progression of their disease. Nearly 70% of individuals with diabetes do not realize they are at increased risk for cardiovascular complications [37]. One possible explanation is that screening and treatment of patients with prediabetes is not universally embraced among family care physicians in the United States [39].

In a national survey of academic family care physicians, attitudes towards prediabetes varied significantly and played a critical role in their treatment and screening behaviors [39]. Within this survey, physicians reported patient insurance coverage, patient ability to modify their lifestyle, and knowledge of treatment options as significant barriers for administering diabetes prevention. Furthermore, physicians who reported negative attitudes towards prediabetes were less likely to believe patients could successfully follow lifestyle changes needed, less likely to agree that prediabetes screening is a high priority for family physicians, and more likely to recommend generic lifestyle changes aimed at reducing cardiovascular disease rather than strategies aimed at lowering blood glucose concentrations [39].

Alternatively, patients could take beneficial steps to control their diabetes after meaningful conversations with their healthcare providers. Evidence-based lifestyle programs targeted at improving diet, exercise, and weight loss can effectively delay or prevent type 2 diabetes [11]. In a 2006 report among American adults who were told they have prediabetes and are at risk for developing type 2 diabetes, 68% attempted weight loss management, 55% increased physical activity or exercise, 60% reduced dietary fat or caloric intake, and 42% engaged in all three activities [40]. Additionally, Japanese participants classified as prediabetic, who experienced comprehensive health checkups including lifestyle education, showed significantly reduced progression from prediabetes to diabetes [41]. Okosun et al. [37] revealed that adults with an increased risk for diabetes significantly improved lifestyle factors associated with weight control, physical activity, and caloric intake. The adoption of these healthy behaviors was significantly greater in individuals who were made aware of their increased type 2 diabetes risk compared to those who were unaware. Healthcare providers should also utilize diabetes educators (e.g., nurses, dietitians, exercise physiologists, etc.) to assist with additional screening opportunities, intervention, and behavioral modifications [28,37]. For instance, it is estimated that each year 19.5 million individuals in the United States visit a dental office without seeing their regular physician [42]. Interestingly, diabetes screening in the dental setting can lead to increased dysglycemia awareness and early disease detection [43].

Other studies have also evaluated the impact of health insurance on undiagnosed type 2 diabetes in Americans. For example, Hogan et al. [44] found that when comparing those without health insurance to a matched cohort of individuals with insurance, the probability of diagnosis for those with insurance was about 14 percentage points higher for diabetes. In addition, cross-sectional data of 2838 Mexican Americans residing on the United States-Mexico border from 2004 to 2014 identified that individuals with diabetes and health insurance had greater odds to be diagnosed compared to those who were uninsured. Within this cohort, individuals with diabetes and Medicaid or Medicare had greater odds to be diagnosed compared to individuals with diabetes and no insurance type [45]. Campbell et al. [46] also revealed that those with no regular care and uninsured had greater odds for unawareness to their prediabetes. Our findings extend the literature by evaluating the role of health insurance type on undiagnosed prediabetes and diabetes in American adults. The results of these studies, along with ours, provide a cluster of evidence for the importance of access to health insurance in avoiding undiagnosed conditions and highlights disparities among health insurance coverage type. 

Undiagnosed prediabetes and type 2 diabetes are also problems in other countries, and nearly half of diabetes cases globally are undiagnosed [47]. For example, 18% of participants from a sub-sample were considered as having undiagnosed type 2 diabetes from the Mexican Health and Aging Study [48]. Moreover, around 590,000 people in the United Kingdom have type 2 diabetes that has not been diagnosed, and globally undiagnosed type 2 diabetes may impact about 179 million people [49]. Alternatively, our findings also showed that a large proportion of Hispanics had no health insurance. Although Hispanics are at an elevated risk for diabetes [50], health disparities with respect to race/ethnicity may exist for health insurance. This underscores the importance of not only continuing to correctly diagnose, prevent, and treat cardiometabolic morbidities on a global level but also addressing healthcare disparities. 

Sex- and age-related differences may help to explain our stratified associations for health insurance type and undiagnosed prediabetes and diabetes. For example, sex hormones largely factor into energy metabolism, body composition, vascular function, and inflammatory responses, and thus, endocrine imbalances lead to adverse cardiometabolic characteristics in women with androgen excess for men with hypogonadism [51]. Likewise, the large heterogeneity in health status and date of diabetes diagnosis in older adults makes treatment recommendations for diabetes especially challenging relative to younger age cohorts [52]. Therefore, environmental and physical considerations between sexes and ages should be acknowledged for population-based studies examining diabetes. 

### Limitations

Some limitations of this study should be noted. Although self-report information is common in large, population-based studies such as the NHANES, self-report information was used in our study to determine undiagnosed prediabetes and type 2 diabetes, thus bias or misreporting may have influenced our findings. The proportions of participants with undiagnosed prediabetes or diabetes in our study may differ from those reported in other studies. Survey estimates of Medicaid enrollment and approximations of those uninsured could have response errors [53]. Smaller sample sizes for certain races/ethnicities limited our ability to conduct additional analyses that were stratified by race/ethnicity. Lack of exogenous variation in the explanatory variable may have implications on causal inference. Opportunities could exist for future investigations to examine the longitudinal associations of health insurance and undiagnosed morbidities, especially because changes to American healthcare policy may have influenced such associations. Future research should also continue to examine how health insurance status impacts other undiagnosed conditions, how health insurance type may differentially influence the treatment of such conditions, and possibilities of additional screening opportunities between men, women, and uninsured individuals of varying ethnicities. 

## 5. Conclusions

This investigation found that having health insurance was associated with decreased odds for undiagnosed prediabetes and type 2 diabetes in American adults. Our findings highlight a large proportion of individuals without health insurance have undiagnosed prediabetes or type 2 diabetes and are therefore probably not managing their blood glucose levels properly. Early detection and management of prediabetes may prevent type 2 diabetes, subsequent complications, and economic burden on healthcare systems. A concerted effort to expand screening criteria and increase access to screening procedures and healthcare should be pursued. 

## Figures and Tables

**Table 1:** Descriptive characteristics of the participants.

Variable	Overall (*n* = 13,029)	Private Health Insurance (*n* = 6801)	Medicaid (*n* = 1125)	Other Health Insurance (*n* = 1426)	No Health Insurance (*n* = 3677)
Age (years)	44.6 ± 12.8	45.4 ± 12.4	44.3 ± 13.5	48.6 ± 12.5	41.6 ± 12.8
Male (*n* (%))	6570 (50.4)	3412 (50.1)	405 (36.0)	699 (49.0)	2054 (55.8)
Race/Ethnicity (*n* (%))					
Black	3092 (23.7)	1501 (22.1)	445 (39.6)	391 (27.4)	755 (20.5)
Hispanic	3790 (29.1)	1441 (21.2)	286 (25.4)	417 (29.2)	1646 (44.8)
White	4801 (36.9)	3035 (44.6)	305 (27.1)	460 (32.3)	1001 (27.2)
Other	1346 (10.3)	824 (12.1)	89 (7.9)	158 (11.1)	275 (7.5)
Obese (*n* (%))	5672 (43.5)	2932 (43.1)	605 (53.7)	690 (48.3)	1445 (39.3)
Below Poverty Threshold (*n* (%))	3665 (28.1)	833 (12.2)	731 (64.9)	491 (34.4)	1610 (43.7)
High School Completed or Equivalent (*n* (%))	9768 (74.9)	5931 (87.2)	664 (59.0)	1034 (72.5)	2139 (58.1)
Self-Rated Health (*n* (%))					
Excellent	1183 (9.1)	762 (11.2)	54 (4.8)	67 (4.7)	300 (8.1)
Very Good	3219 (24.7)	2151 (31.6)	154 (13.7)	268 (18.8)	646 (17.5)
Good	5350 (41.1)	2794 (41.1)	404 (35.9)	566 (39.7)	1586 (43.1)
Fair	2741 (21.0)	964 (14.2)	375 (33.3)	405 (28.4)	997 (27.1)
Poor	536 (4.1)	130 (1.9)	138 (12.3)	120 (8.4)	148 (4.0)
Place of Healthcare (*n* (%))					
ER, Outpatient Department, Other	849 (6.5)	233 (3.4)	96 (7.6)	145 (10.2)	375 (10.2)
Clinic or Health Center	2748 (21.1)	1068 (15.7)	349 (31.0)	416 (29.2)	915 (24.9)
Doctor’s Office or HMO	7006 (53.8)	4782 (70.3)	594 (52.8)	731 (51.2)	899 (24.4)
None	2426 (18.6)	718 (10.6)	86 (7.6)	134 (9.4)	1488 (40.5)
Undiagnosed Prediabetes (*n* (%))	5976 (45.8)	3067 (45.1)	452 (40.1)	627 (43.9)	1830 (49.7)
Undiagnosed Diabetes (*n* (%))	897 (6.8)	418 (6.1)	80 (7.1)	111 (7.7)	288 (7.8)
Undiagnosed Prediabetes or Diabetes (*n* (%))	6873 (52.7)	3485 (51.2)	532 (47.2)	738 (51.7)	2118 (57.6)

ER = emergency room; HMO = health maintenance organization.

**Table 2:** Associations of health insurance type and undiagnosed prediabetes and type 2 diabetes.

Variable	Odds Ratio	95% CI	Marginal Effect |(%)|
*Undiagnosed Prediabetes* ^†^			
Medicaid	0.78	0.67, 0.90	6.0 ± 0.3
Private Insurance	0.87	0.79, 0.95	3.3 ± 0.2
Other Insurance	0.84	0.73, 0.95	4.1 ± 0.2
*Undiagnosed Type 2 Diabetes* ^†^			
Medicaid	0.86	0.65, 1.13	0.9 ± 0.6
Private Insurance	0.82	0.67, 0.99	1.2 ± 0.7
Other Insurance	0.83	0.65, 1.07	1.1 ± 0.6
*Undiagnosed Prediabetes or Type 2 Diabetes* ^†^			
Medicaid	0.75	0.65, 0.86	6.8 ± 0.4
Private Insurance	0.82	0.74, 0.90	4.6 ± 0.3
Other Insurance	0.80	0.70, 0.91	5.2 ± 0.3

^†^ Reference: No Insurance. Each model was adjusted for the place of healthcare, self-rated health, race, obesity, sex, age, educational achievement, and poverty threshold. CI = confidence interval.

**Table 3:** Associations of health insurance type and undiagnosed prediabetes and type 2 diabetes stratified by sex and age group.

Variable	Odds Ratio	95% CI	Marginal Effect |(%)|
**Males**			
*Undiagnosed Prediabetes* ^†^			
Medicaid	0.66	0.53, 0.83	10.0 ± 0.3
Private Insurance	0.85	0.75, 0.97	3.9 ± 0.1
Other Insurance	0.82	0.68, 0.98	4.9 ± 0.1
*Undiagnosed Type 2 Diabetes* ^†^			
Medicaid	0.90	0.58, 1.38	0.7 ± 0.4
Private Insurance	0.84	0.65, 1.08	1.1 ± 0.6
Other Insurance	0.77	0.54, 1.09	1.7 ± 0.1
*Undiagnosed Prediabetes or Type 2 Diabetes* ^†^			
Medicaid	0.64	0.51, 0.80	10.4 ± 0.6
Private Insurance	0.81	0.70, 0.92	5.0 ± 0.3
Other Insurance	0.76	0.63, 0.91	6.4 ± 0.4
**Females**			
*Undiagnosed Prediabetes* ^†^			
Medicaid	0.88	0.73, 1.07	2.8 ± 0.2
Private Insurance	0.91	0.78, 1.05	2.2 ± 0.2
Other Insurance	0.89	0.74, 1.08	2.5 ± 0.2
*Undiagnosed Type 2 Diabetes* ^†^			
Medicaid	0.80	0.55, 1.16	1.2 ± 0.8
Private Insurance	0.77	0.58, 1.03	1.4 ± 0.9
Other Insurance	0.90	0.63, 1.28	0.6 ± 0.4
*Undiagnosed Prediabetes or Type 2 Diabetes* ^†^			
Medicaid	0.84	0.69, 1.01	4.1 ± 0.2
Private Insurance	0.85	0.74, 0.99	3.7 ± 0.2
Other Insurance	0.87	0.72, 1.05	3.1 ± 0.2
**Aged 18–44 Years**			
*Undiagnosed Prediabetes* ^†^			
Medicaid	0.98	0.80, 1.21	0.3 ± 0.1
Private Insurance	1.05	0.85, 1.30	2.4 ± 0.2
Other Insurance	0.89	0.78, 1.03	1.1 ± 0.1
*Undiagnosed Type 2 Diabetes* ^†^			
Medicaid	0.83	0.51, 1.36	0.7 ± 0.6
Private Insurance	0.67	0.48, 0.93	1.6 ± 1.3
Other Insurance	0.84	0.52, 1.36	0.7 ± 0.6
*Undiagnosed Prediabetes or Type 2 Diabetes* ^†^			
Medicaid	0.94	0.76, 1.16	1.4 ± 0.1
Private Insurance	0.83	0.72, 0.96	4.0 ± 0.5
Other Insurance	1.02	0.82, 1.26	0.5 ± 0.1
**Aged 45–64 Years**			
*Undiagnosed Prediabetes* ^†^			
Medicaid	0.70	0.57, 0.87	8.3 ± 0.5
Private Insurance	0.88	0.77, 1.01	2.9 ± 0.2
Other Insurance	0.83	0.70, 0.99	4.3 ± 0.3
*Undiagnosed Type 2 Diabetes* ^†^			
Medicaid	0.93	0.66, 1.30	0.6 ± 0.2
Private Insurance	0.91	0.72, 1.15	0.7 ± 0.3
Other Insurance	0.90	0.67, 1.21	0.8 ± 0.3
*Undiagnosed Prediabetes or Type 2 Diabetes* ^†^			
Medicaid	0.69	0.56, 0.85	8.7 ± 0.6
Private Insurance	0.85	0.74, 0.98	3.7 ± 0.2
Other Insurance	0.80	0.67, 0.95	5.1 ± 0.3

^†^ Reference: No Insurance. Each model was adjusted for the place of healthcare, self-rated health, race, obesity, sex (when applicable), age, educational achievement, and poverty threshold. CI = confidence interval.

## Appendix A

**Table A1:** Means and 95% confidence intervals for the descriptive characteristics of the participants.

Variable	Overall	Private Health Insurance	Medicaid	Other Health Insurance	No Health Insurance
Age (Years)	44.6 (44.3, 44.8)	45.4 (45.1, 45.7)	44.3 (43.5, 45.0)	48.6 (48.0, 49.3)	41.6 (41.2, 42.0)
Male (%)	50.4 (49.5, 51.2)	50.1 (48.9, 51.3)	36.0 (33.2, 38.8)	49.0 (46.4, 51.6)	55.8 (54.2, 57.4)
Race (%)					
Black	23.7 (23.0, 24.4)	22.1 (21.0, 23.0)	39.6 (36.7, 42.4)	27.4 (25.1, 29.7)	20.5 (19.2, 21.8)
Hispanic	29.1 (28.3, 29.8)	21.2 (20.2, 22.1)	25.4 (22.8, 27.9)	29.2 (26.8, 31.6)	44.8 (43.1, 46.3)
White	36.9 (36.0, 37.6)	44.6 (43.4, 45.8)	27.1 (24.5, 29.7)	32.3 (29.8, 34.6)	27.2 (25.7, 28.6)
Other	10.3 (9.8, 10.8)	12.1 (11.3, 12.8)	7.9 (6.3, 9.4)	11.1 (9.4, 12.7)	7.5 (6.6, 8.3)
Obese (%)	43.5 (42.6, 44.3)	43.1 (41.9, 44.2)	53.7 (50.8, 56.6)	48.3 (45.7, 50.9)	39.3 (37.7, 40.8)
Below Poverty Threshold (%)	28.1 (27.3, 28.9)	12.2 (11.4, 13.0)	64.9 (62.1, 67.7)	34.4 (31.9, 36.9)	43.7 (42.1, 45.3)
High School Completed or Equivalent (%)	74.9 (74.2, 75.7)	87.2 (86.4, 88.0)	59.0 (56.1, 61.9)	72.5 (70.1, 74.8)	58.1 (56.5, 59.7)
Self-Rated Health (%)					
Excellent	9.1 (8.5, 9.5)	11.2 (10.4, 11.9)	4.8 (3.5, 6.0)	4.7 (3.6, 5.8)	8.1 (7.2, 9.0)
Very Good	24.7 (23.9, 25.4)	31.6 (30.5, 32.7)	13.7 (11.6, 15.7)	18.8 (16.7, 20.8)	17.5 (16.3, 18.8)
Good	41.1 (40.2, 41.9)	41.1 (33.9, 42.2)	35.9 (33.1, 38.7)	39.7 (37.1, 42.2)	43.1 (41.5, 44.7)
Fair	21.0 (20.3, 21.7)	14.2 (13.3, 15.0)	33.3 (30.5, 36.0)	28.4 (26.0, 30.7)	27.1 (25.6, 28.5)
Poor	4.1 (3.7, 4.4)	1.9 (1.5, 2.2)	12.3 (10.3, 14.1)	8.4 (6.9, 9.8)	4.0 (3.3, 4.6)
Place of Healthcare (%)					
ER, Outpatient Department, Other	6.5 (6.0, 6.9)	3.4 (2.9, 3.8)	7.6 (6.9, 10.1)	10.2 (8.6, 11.7)	10.2 (9.2, 11.1)
Clinic or Health Center	21.1 (20.3, 21.7)	15.7 (14.8, 16.5)	31.0 (28.3, 33.7)	29.2 (26.8, 31.5)	24.9 (23.4, 26.2)
Doctor’s Office or HMO	53.8 (52.9, 54.6)	70.3 (69.2, 71.4)	52.8 (49.8, 55.7)	51.2 (48.6, 53.8)	24.4 (23.0, 25.8)
None	18.6 (17.9, 19.2)	10.6 (9.8, 11.2)	7.6 (6.0, 9.2)	9.4 (7.8, 10.9)	40.5 (38.8, 42.0)
Undiagnosed Prediabetes (%)	45.8 (45.0, 46.7)	45.1 (43.9, 46.2)	40.1 (37.3, 43.0)	43.9 (41.3, 46.5)	49.7 (48.1, 51.3)
Undiagnosed Type 2 Diabetes (%)	6.8 (6.4, 7.3)	6.1 (5.5, 6.7)	7.1 (5.6, 8.6)	7.7 (6.3, 9.1)	7.8 (6.9, 8.7)
Undiagnosed Prediabetes or Type 2 Diabetes (%)	52.7 (51.8, 53.6)	51.2 (50.0, 52.4)	47.2 (44.3, 50.2)	51.7 (49.1, 54.3)	57.6 (56.0, 59.2)

ER = emergency room; HMO = health maintenance organization.

**Table A2:** Associations of health insurance type and undiagnosed prediabetes and type 2 diabetes stratified by educational achievement and race/ethnicity.

Variable	Odds Ratio	95% Confidence Interval
**Did Not Graduate High School**		
*Undiagnosed Prediabetes* ^†^		
Medicaid	0.71	0.56, 0.90
Private Insurance	0.82	0.68, 0.99
Other Insurance	0.80	0.63, 1.02
*Undiagnosed Type 2 Diabetes* ^†^		
Medicaid	1.04	0.68, 1.58
Private Insurance	0.82	0.58, 1.16
Other Insurance	1.07	0.70, 1.63
*Undiagnosed Prediabetes or Type 2 Diabetes* ^†^		
Medicaid	0.72	0.57, 0.90
Private Insurance	0.76	0.63, 0.92
Other Insurance	0.81	0.64, 1.03
**At Least A High School Graduate or Equivalent**		
*Undiagnosed Prediabetes* ^†^		
Medicaid	0.82	0.68, 1.00
Private Insurance	0.89	0.80, 1.00
Other Insurance	0.86	0.74, 1.01
*Undiagnosed Type 2 Diabetes* ^†^		
Medicaid	0.73	0.50, 1.07
Private Insurance	0.80	0.64, 1.01
Other Insurance	0.73	0.54, 1.00
*Undiagnosed Prediabetes or Type 2 Diabetes* ^†^		
Medicaid	0.77	0.64, 0.93
Private Insurance	0.85	0.76, 0.95
Other Insurance	0.81	0.69, 0.94
**Not White**		
*Undiagnosed Prediabetes* ^†^		
Medicaid	0.81	0.68, 0.96
Private Insurance	0.91	0.81, 1.03
Other Insurance	0.80	0.68, 0.94
*Undiagnosed Type 2 Diabetes* ^†^		
Medicaid	0.96	0.70, 1.31
Private Insurance	0.88	0.70, 1.10
Other Insurance	0.93	0.70, 1.25
*Undiagnosed Prediabetes or Type 2 Diabetes* ^†^		
Medicaid	0.80	0.68, 0.95
Private Insurance	0.88	0.78, 0.99
Other Insurance	0.79	0.67, 0.92
**White**		
*Undiagnosed Prediabetes* ^†^		
Medicaid	0.75	0.57, 0.99
Private Insurance	0.82	0.70, 0.97
Other Insurance	0.97	0.77, 1.22
*Undiagnosed Type 2 Diabetes* ^†^		
Medicaid	0.59	0.33, 1.07
Private Insurance	0.71	0.50, 1.00
Other Insurance	0.65	0.40, 1.05
*Undiagnosed Prediabetes or Type 2 Diabetes* ^†^		
Medicaid	0.67	0.50, 0.88
Private Insurance	0.77	0.65, 0.91
Other Insurance	0.89	0.70, 1.13

^†^ Reference: No Insurance. Each model was adjusted for the place of healthcare, self-rated health, race (when applicable), obesity, sex, age, educational achievement (when applicable), and poverty threshold.
